# Nutrition and Supplements during Pregnancy: A Vital Component in Building the Health and Well-Being of Both the Mother and the Developing Baby

**DOI:** 10.3390/nu15153395

**Published:** 2023-07-31

**Authors:** Yushu Qin, Linglin Xie

**Affiliations:** Department of Nutrition, Texas A & M University, College Station, TX 77843, USA; yushu@tamu.edu

Maternal health is of the utmost importance during pregnancy, not just for the mother but also for the developing fetus. While pregnant women focus on their diet to ensure the growth and well-being of their unborn child, the significance of essential nutrients and supplements in improving outcomes and reducing risks remains uncertain. Inadequate or excessive nutrition during the prenatal and early stages of life can disrupt normal growth and development, potentially leading to future health problems for the offspring. To address this issue, our Special Issue comprises eight original research articles, one systematic review, and one review article that highlight the crucial role of nutrients and supplements during pregnancy. These research pieces aim to inform readers about the potential benefits, important considerations, and far-reaching implications of proper nutrition for the well-being of both mothers and children.

Pregnancy brings significant changes to the body, necessitating increased nutritional support for both the developing fetus and the mother’s health. Essential nutrients such as vitamins, minerals, and omega-3 fatty acids are vital to meet these demands. However, the study by Koivuniemi et al. suggests that many people lack awareness of the recommended nutrient intake levels, and compliance with them is lower than expected [1]. Therefore, it is crucial to enhance education and awareness of proper nutrition during pregnancy to ensure a healthier pregnancy journey for both the mother and the baby. By providing accurate information and guidance, expectant mothers can bridge this knowledge gap and make informed decisions about their diet and supplement choices.

The increasing prevalence of pregnancy complications, including gestational diabetes mellitus (GDM) and preeclampsia (PE), is a cause for concern due to the potential health risks for both mothers and infants. Although the exact causes of these complications remain unclear, research suggests that the placenta’s pathological state plays a critical role. Yu et al.’s review provides an in-depth overview of PPARγ’s role in placental pathophysiology and explores the potential of PPARγ ligands as a treatment option for managing pregnancy complications [2]. The study highlights the importance of controlling blood glucose levels as a crucial component in managing GDM and PE. Similarly, a meta-analysis by Dingena et al. supports the significance of nutritional supplements, diet, and exercise in managing blood glucose levels in GDM, demonstrating promising positive effects on glycemia measures such as fasting plasma glucose (FPG), post-prandial glucose (PPG), and the Homeostatic Model of Assessment (HOMA-IR) [3]. HOMA-IR demonstrated the most significant effect sizes and the least heterogeneity, receiving the best GRADE (Grading of Recommendations Assessment, Development and Evaluation) rating among these measures. For future randomized controlled trials (RCTs), considering HOMA-IR as an outcome in the study design and possibly combining different intervention types could be beneficial. However, it is essential to note that evidence for the effectiveness of these interventions in other types of diabetes, such as pre-existing diabetes, remains limited, indicating the need for further research in these specific populations.

Folic acid is widely recognized as a crucial supplement for promoting healthy fetal development and reducing the risk of neural tube defects during pregnancy and is therefore recommended by health practitioners. However, studies have produced varying conclusions regarding its association with the development of GDM, leading to inconclusive findings. In our Special Issue, Chen et al. conducted a significant longitudinal study involving 24,429 pregnant Chinese women to investigate the potential link between folic acid supplementation in early pregnancy and the risk of GDM [4]. The study’s findings suggested that folic acid supplementation may act as a protective factor against the risk of GDM, particularly in women with a normal pre-pregnancy BMI. However, the study did not observe any specific dose or duration-related associations between folic acid supplementation and the risk of GDM. Notably, two prospective pregnancy cohorts performed in Australia investigating the impact of folic acid fortification unveiled changes in hormonal profiles, raising concerns regarding the appropriateness of folic acid supplementation and the potential risks related to insulin resistance and gestational diabetes in specific populations [5]. These results highlight the complexities in understanding the relationship between folic acid supplementation and GDM risk. While some studies have suggested a potential association, the evidence remains inconclusive and may depend on various factors. Further research is necessary to understand how folic acid supplementation may impact the development of GDM, identify specific risk factors, and determine potential benefits for different populations. Pregnant women should consult healthcare professionals to make informed decisions about folic acid supplementation during pregnancy based on their individual circumstances.

Apart from nutrients, eating habits before and after pregnancy significantly affect pregnancy outcomes. A preliminary investigation into the dietary practices of pregnant women revealed that many women aim to improve their lifestyle during pregnancy. However, the study also highlighted the emergence of unhealthy orthorexia nervosa tendencies in some cases. Although most participants reported dietary improvements, a small number of them exhibited pica tendencies. The study also emphasized the prevalence of severe nausea and emesis during pregnancy, highlighting the need to identify and address disturbed eating behaviors to ensure optimal pregnancy outcomes [6].

Research has indicated that food and supplement choices have a significant impact on intergenerational effects. Women with gluten-related diseases are at a higher risk of infertility [7]. Lee et al. conducted a study focusing on the effects of gliadin on oocyte quality in Caenorhabditis elegans. The research revealed that a high intake of gliadin peptide, a major component of gluten, had adverse effects on oocyte quality, resulting in chromosomal abnormalities and mitochondrial oxidative stress. This led to increased embryonic lethality. However, the study also suggested that these detrimental effects could be mitigated through antioxidant treatment, highlighting potential intervention strategies relying on antioxidants [8].

During pregnancy, the intake of fatty acids is a crucial consideration as it significantly impacts the health outcomes of both the mother and the fetus. Essential fatty acids and long-chain polyunsaturated fatty acids are deemed beneficial for fetal development and the birthing process. Omega-3 dietary supplementation appears to be the most efficient method to enhance levels of circulating long-chain polyunsaturated fatty acids, rather than solely relying on increased fish and seafood consumption [9]. Short-chain fatty acids like butyrate and propionate have also shown favorable effects in addition to long-chain fatty acids. These short-chain fatty acids are commonly produced as metabolites by gut microbiota. Research conducted in rats indicates that supplementing with butyrate and propionate during pregnancy and lactation prevented hypertension in offspring that may arise due to maternal high-fructose intake during pregnancy [10]. This highlights early life postbiotic supplementation as a potential strategy for enhancing the health status of both mothers and their children.

Recent animal studies have demonstrated a concerning association between maternal high-fat diet consumption and an increased risk of non-alcoholic fatty liver disease (NAFLD) in offspring. This link is attributed to disruptions in the methionine cycle [11]. However, there is encouraging news from a study by Hu et al., conducted on mice, which found that maternal one-carbon supplementation effectively reduces the risk of NAFLD by restoring the normal state of the methionine cycle [12]. The study also identified crucial CpG sites of genes related to liver lipid metabolism, highlighting the significance of DNA methyltransferase (DNMT) activity in programming the offspring’s liver lipid metabolism. Further research is needed to understand the epigenetic mechanisms underlying the connection between maternal nutrition and offspring metabolism. Nevertheless, this study supports the possibility of implementing nutritional strategies for expectant mothers to prevent NAFLD in their offspring.

While there is already a significant amount of knowledge on nutritional supplementation during pregnancy, certain aspects require further investigation. Key areas that need continued research include evaluating the long-term effects of various supplements, determining the optimal dosage and duration for specific nutrients, and identifying potential risks associated with supplementation. Additionally, understanding the interactions between different nutrients when taken together is critical for providing comprehensive and accurate dietary recommendations for expectant mothers.

To ensure safe and informed decision-making, it is essential to promote education and raise awareness among healthcare professionals, expectant mothers, and the public regarding supplement use during pregnancy. By disseminating accurate information, individuals can make well-informed choices, thus improving maternal and fetal health outcomes. Addressing these research gaps and fostering awareness can better support the health and well-being of pregnant women and their developing babies.
